# Favorable effect of bortezomib in dense deposit disease associated with monoclonal gammopathy: a case report

**DOI:** 10.1186/s12882-018-0905-6

**Published:** 2018-05-03

**Authors:** Shuma Hirashio, Ayaka Satoh, Takahiro Arima, Kouichi Mandai, Tadasuke Awaya, Kumi Oshima, Shigeo Hara, Takao Masaki

**Affiliations:** 1 Department of Nephrology, Hiroshima University Hospital, 1-2-3 Kasumi, Minami-ku, Hiroshima, 7348551 Japan; 2 Department of Nephrology, National Hospital Organization Higashihiroshima Medical Center, Hiroshima, Japan; 3 Department of Diagnostic Pathology, National Hospital Organization Higashihiroshima Medical Center, Hiroshima, Japan; 4 Department of Hematology, Hiroshima University Hospital, Hiroshima, Japan; 5 Department of Diagnostic Pathology, Kobe University Graduate School of Medicine, Kobe, Japan

**Keywords:** C3 glomerulopathy, Complement, Dense deposit disease, Monoclonal gammopathy of renal significance, Monoclonal gammopathy of undetermined significance

## Abstract

**Background:**

Complement component 3 (C3) glomerulopathy, which includes dense deposit disease (DDD) and C3 glomerulonephritis, is caused by dysregulation of the alternative complement pathway. In most cases, C3 glomerulopathy manifests pathologically with membranoproliferative glomerulonephritis-like features. An association between C3 glomerulopathy and monoclonal gammopathy was recently reported in several cases, raising the possibility that C3 glomerulopathy is the underlying pathological process in monoclonal gammopathy of renal significance.

**Case presentation:**

We herein report a case of monoclonal gammopathy-induced DDD that improved histologically and clinically with chemotherapy including bortezomib. Our case is the first in which treatment response can be linked to the histological response. Potential pathological insights are also discussed.

**Conclusions:**

Rapid and efficient chemotherapy has the potential to limit renal damage in monoclonal gammopathy-associated DDD.

## Background

The complement system regulates innate immune responses that eliminate microbial pathogens. Activation of the complement pathway is strictly controlled by the combined effects of various inhibitory factors to prevent its uncontrolled activation and inappropriate immunological responses to human tissues [1, 2]. Complement activation is also associated with the development of various types of glomerulonephritis. In particular, the complement system plays a pivotal role in the development of membranoproliferative glomerulonephritis (MPGN) [3]. Complement component 3 (C3) glomerulopathy, which includes dense deposit disease (DDD) and C3 glomerulonephritis, is a recently described disease entity caused by dysregulation of the alternative complement pathway [4–8]. Factors known to activate complement are presumably associated with the development of C3 glomerulopathy as part of a second “hit.”

A case of MPGN resulting from multiple myeloma-induced monoclonal gammopathy was recently reported [9]. Monoclonal gammopathy of renal significance (MGRS) is a distinct renal disorder caused by monoclonal immunoglobulins in patients with detectable serum or urine monoclonal gammopathy [10]. Although abnormal plasma cell populations have poor proliferative capacity, MGRS is associated with the presence of so-called dangerous small B-cell clones, which produce an M protein that is highly likely to be deposited in tissue [11]. The significance of early therapeutic intervention for underlying disease in MGRS has been highlighted recently [10]. In addition to MPGN, reports of patients with concomitant C3 glomerulopathy and monoclonal gammopathy of unknown significance (MGUS) offer valuable insights into the relationship between these two diseases [12, 13]. We herein report our experience with a patient diagnosed with monoclonal gammopathy-associated DDD based on clinical features and pathological findings of renal biopsy. The patient was treated with induction therapy using a combination of bortezomib and dexamethasone (BD therapy). After 13 courses of BD therapy the patient had improvement in serum complement level and proteinuria, as well as biopsy-confirmed histological resolution of DDD.

## Case presentation

A 52-year-old man was referred to our hospital for detailed evaluation of renal dysfunction. The patient had been evaluated at another medical institution for edema in both lower limbs a few months before admittance to our hospital. At that time, he had a urine protein level exceeding 1 g/gCr and an estimated glomerular filtration rate (eGFR) of 40 mL/min/1.73 m^2^.

The patient’s medical history and family history were unremarkable. He had no history of infection, drug therapy, or other potential causative factors for renal dysfunction or proteinuria. Physical examination on admission revealed a body weight of 69.8 kg (a 2-kg increase from his usual weight) and symmetrical pitting edema in both lower limbs. Blood pressure was 166/88 mmHg. Initial laboratory evaluation (Table 1) revealed elevated serum creatinine (1.65 mg/dL) and an eGFR of 36.1 mL/min/1.73 m^2^. The patient’s complement activity was high at 12.6 U/mL as determined with the total complement activity (CH50) test. Hypocomplementemia was noted, with low C3 and C4 levels (43 and 6 mg/dL, respectively). Urinalysis revealed 3+ proteinuria, 20 to 29 red blood cells per high-power field, and 5 to 9 fatty/waxy casts per whole field. Urine protein/creatinine ratio was 1.61 g/gCr with a spot measurement. Furthermore, M protein was detected on urinalysis; a separate blood test revealed the presence of λ light chains. The plasma cell count in the bone marrow was 2.4%, which excluded the diagnosis of multiple myeloma. The chromosomal pattern in the bone marrow was normal. Bone positron emission tomography revealed no abnormal uptake. Although the patient was diagnosed with MGUS, marked organ dysfunction localized to the kidneys was determined to reflect a pathological diagnosis of MGRS.

**Table 1:** Initial laboratory findings

Parameter	Value	Reference range
(Urine)		
pH	5.5	5.0–6.5
red blood cell (/HPF)	20–29	< 5
fatty casts (/WF)	5–9	negative
waxy casts (/WF)	5–9	negative
Urine protein/creatinine ratio (g/g)	0.61	< 0.15
M protein	positive	negative
(Blood)		
Leukocyte count (/μL)	7400	4500–9000
Hemoglobin (g/dL)	12.3	13.6–17.0
Platelet count (× 10^4^/μL)	16.3	14–36
Urea nitrogen (mg/dL)	24.5	8.0–22.0
Creatinine (mg/dL)	1.65	0.60–1.10
Uric acid (mg/dL)	8.6	3.6–7.0
Total protein (g/dL)	5.9	6.7–8.3
Albumin (g/dL)	3.7	4.0–5.0
Lactate dehydrogenase (IU/L)	223	119–229
Sodium (mEq/L)	140	138–146
Potassium (mEq/L)	5.3	3.6–4.9
Chloride (mEq/L)	108	99–109
Corrected serum calcium (mg/dL)	8.8	8.6–10.4
Phosphate (mg/dL)	3.5	2.5–4.7
C-reactive protein (mg/dL)	0.07	< 0.30
CH50 (CH50/mL)	12.6	25–48
C3 (mg/dL)	43	65–135
C4 (mg/dL)	6	13–35
IgG (mg/dL)	889	870–1700
IgA (mg/dL)	271	110–410
IgM (mg/dL)	39	33–190
IgE (IU/mL)	9.0	< 269
light chain type κ(mg/L)	51.0	2.42–18.92
type λ(mg/L)	517.0	4.44–26.18
Cryoglobulin	negative	negative
Anti Factor H antibody (AU/mL)	111.1	< 8.0
sC5b-9 levels (μg/mL)	0.32	0.32–0.72^a^

*HPF* high-power field, *WF* whole field, *CH50* 50% hemolytic complement activity, *IgG* immunoglobulin G, *IgA* immunoglobulin A, *IgM* immunoglobulin M, *IgE* immunoglobulin E

^a^Compared with normal control

**Fig. 1:**
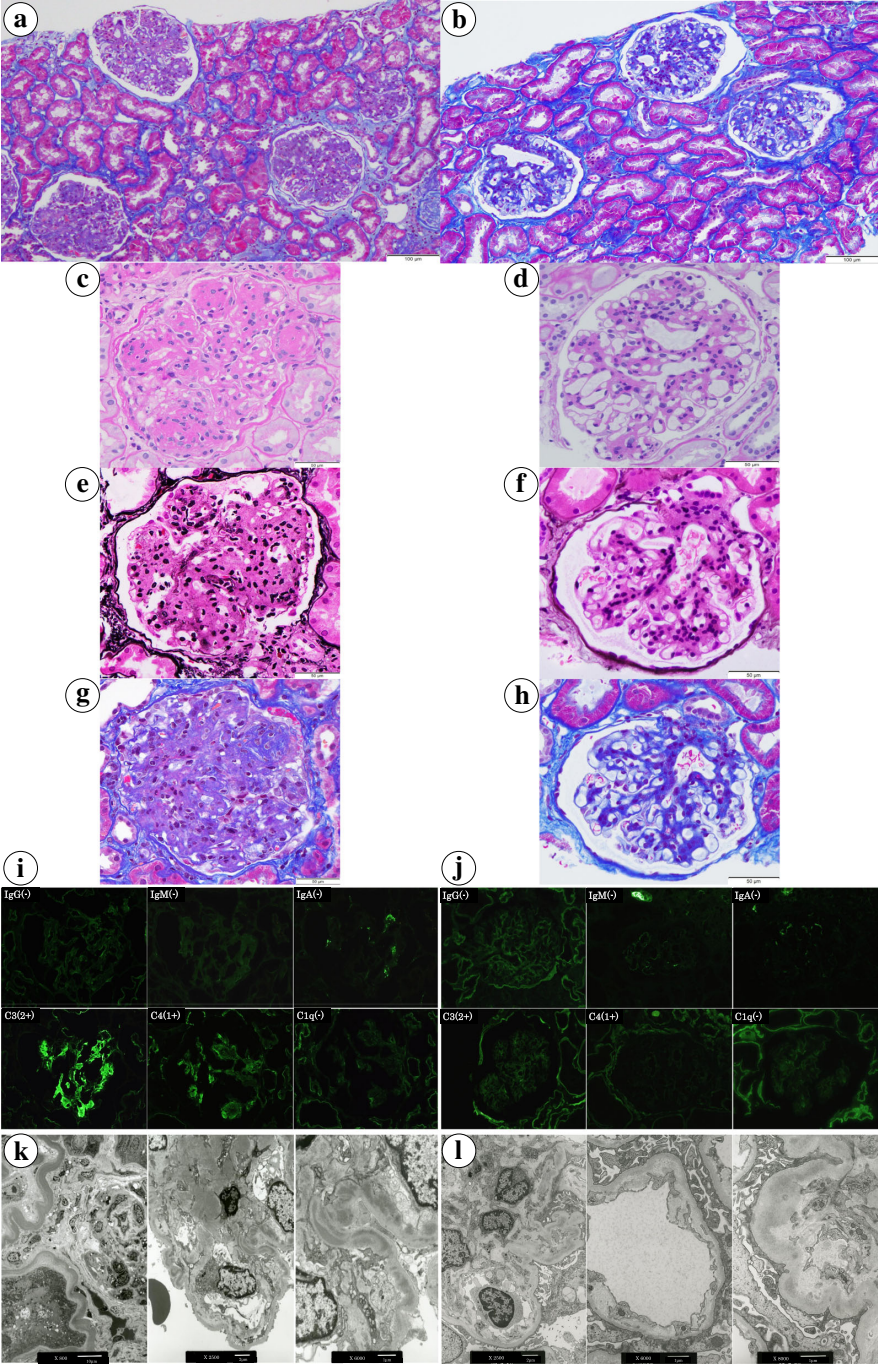
Histological examination of renal biopsy specimens before (**a**, **c**, **e**, **g**, **i**, and **k**) and after (**b**, **d**, **f**, **h**, **j**, and **l**) bortezomib/dexamethasone therapy. In the first biopsy, glomeruli show lobular configuration with nodular formation and endocapillary hypercellularity (**a**, **c**, **e**, and **g**). The second biopsy reveals moderate mesangial cell proliferation and matrix increase without nodules (**b**, **d**, **f**, and **h**). A significant reduction in deposits is seen in all samples after treatment. Staining for C3 (**j**) is decreased after treatment. **a** and **b**. Masson trichrome staining at low magnification (×200). The scale bar represents 100 μm. **c** and **d**. Periodic acid-Schiff staining of glomeruli (×400). The scale bar represents 50 μm. **e** and **f**. Periodic acid–methenamine silver staining of glomeruli (×400). The scale bar represents 50 μm. **g** and **h**. Masson trichrome staining of glomeruli (×400). The scale bar represents 50 μm. **i** and **j**. Immunofluorescent images of frozen sections stained for immunoglobulins and complement. The staining for immunoglobulin was negative. The staining for C3 was strongly positive before BD therapy but negative after treatment. **k** and **l**. Electron microscopic findings. Before BD therapy (**k**), band-like areas of dense deposits along the basement membrane are clearly noticeable. Large deposits are also observed in the mesangial matrix. Deposits are less prominent after treatment (**l**). The scale bars at the bottom of the images represent respective numerical values

Global sclerosis was observed in six glomeruli among a total of 71 glomeruli collected at biopsy. The remaining glomeruli were lobulated, showing a marked increase in mesangial matrix and mesangial cell proliferation. Nodular lesions were also observed, accompanied by mild intracapillary hypercellularity. Tubular atrophy and interstitial fibrosis were distributed in a patchy pattern. Lymphocytic infiltration was localized, surrounding the sclerotic glomeruli. Direct fast scarlet staining was negative, indicating the absence of amyloid deposition. Immunostaining of paraffin sections was negative for κ and λ light chains. Immunofluorescence examination of frozen sections revealed marked deposition of C3 in the mesangial region and weak C4 staining; no immunoglobulin deposition was identified. Electron microscopy revealed linear band-like electron densities within the lamina densa, with alternating thinner segments. Similar dense deposits were observed along the basement membrane of Bowman’s capsule and the renal tubules, in addition to the mesangial matrix. At higher magnification, the electron-dense deposits showed a fine granular pattern and lacked tubular or fibrillary substructures. On the basis of the collective clinical and pathological presentation, the patient was diagnosed with DDD (Fig. 1 a, c, e, g, i, k).

**Fig. 2:**
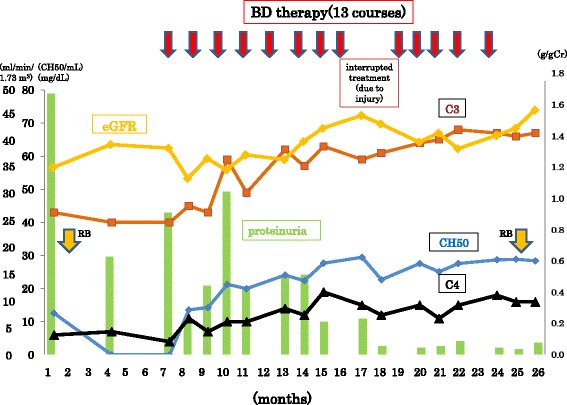
Clinical course. The levels of serum complement proteins (C3, C4), CH50, and eGFR are presented. The green bar represents the urine protein/creatinine ratio. BD therapy, bortezomib and dexamethasone therapy; eGFR, estimated glomerular filtration rate; CH50, 50% hemolytic complement activity; RB, renal biopsy

Because M protein was detected in both the serum and urine, the patient’s pathological diagnosis was MGRS. The serum level of anti-factor H antibody was markedly elevated at 111.1 AU/mL, an elevation attributed to the presence of nephritogenic monoclonal λ light chains [14]. The C5b-9 complex level remained within the normal range. With a diagnosis of MGUS as the underlying cause of DDD, BD therapy (bortezomib, 1.3 mg/m^2^, 2.0 mg/body and dexamethasone, 20 mg) was initiated based on the established treatment protocol for multiple myeloma. In addition, we used atorvastatin, vitamin D, and irbesartan to decrease the patient’s urine protein. As shown in Fig. 2, the patient’s CH50 level gradually increased during treatment, eventually reaching the normal range. His urine protein level decreased to less than 0.5 g/gCr after eight courses of BD therapy. Although not fully normalized, the patient’s eGFR level gradually increased to 49.6 mL/min/1.73 m^2^ after 13 courses of BD therapy.

A second renal biopsy was performed after 13 courses of BD therapy (18 months after the first course) to evaluate the therapeutic effects histologically. Light microscopy revealed an apparent decrease in the mesangial matrix and lobular accentuation of the glomeruli (Fig. 1 b, d, f, h, j, and l). No nodular lesions or mesangiolysis was observed. All immunofluorescence staining was negative. Electron microscopy revealed a decrease in the amount of dense deposits within the basement membrane as well as in the mesangial area. The anti-factor H antibody level had decreased to less than 3.9 AU/mL. These results suggest that treatment of MGUS with BD therapy contributed to the histological resolution of DDD and the serological improvement in complement activation.

## Discussion and conclusions

There is one previous report of treatment response in a patient with C3 glomerulopathy and MGRS who was treated with bortezomib and dexamethasone [15]. However, that report did not describe the link between therapeutic response and histological changes. Our case is the first in which treatment response can be linked to the histological response.

DDD was originally referred to as MPGN type II, which was pathologically defined as the presence of band-like electron-dense deposits along the basement membrane. DDD was recently reclassified as a novel disease entity characterized by dysregulation of the alternative complement pathway [9, 16]. The laboratory findings in our case, including hypocomplementemia and the presence of anti-factor H antibody, suggest that dysregulation of complement activation was the primary pathology. Our patient had low C3 and C4 values, indicating activation of both the classical and alternative complement pathways. In general, DDD does not cause decreased C4. The association between low C4 and the pathology of DDD remains unknown. Although several treatment approaches, including plasmapheresis [5], steroid therapy [17], and renal transplantation [18], have been reported in DDD patients, there are currently no established treatment strategies.

In recent years, monoclonal gammopathy has emerged as one underlying cause of DDD [13]. In the present case, the direct association between disease development and the presence of monoclonal immunoglobulins was supported by the observed improvement in monoclonal protein levels, hypocomplementemia, and pathological findings following BD therapy. Monoclonal λ light chains isolated from patients with MPGN have been shown to act as autoantibodies against factor H [14]. Factor H protects autologous cells from complement-mediated cytotoxicity. The monoclonal λ light chains abnormally produced by plasma cells act as autoantibodies against factor H. This anti-factor H antibody is thought to initiate a sustained complement activation reaction. After BD therapy in our patient, the monoclonal λ chain levels fell to below the sensitivity of the assay, as did anti-factor H antibody levels. This decrease in anti-factor H antibody indicates lack of DDD disease progression. Similarly, the elimination of abnormal plasma cell clones with BD therapy suppressed the production of monoclonal immunoglobulins, which was likely the cause of dysregulation of the alternative complement pathway via inhibition of factor H activity. Bridoux et al. reported that the structural characteristics of monoclonal immunoglobulins, particularly in hypervariable regions, greatly influenced the development of MGRS [19]. We therefore hypothesize that the characteristic tertiary structure of abnormal immunoglobulins in the present case might have contributed to inhibition of factor H, ultimately resulting in DDD.

Chemotherapy is not recommended for MGUS, which is considered a benign hematological disorder that is not associated with organ damage [10]. However, in some patients with MGUS, nephrotoxic monoclonal immunoglobulins preferentially affect specific compartments of the renal tissue, including the glomeruli and renal tubules, leading to characteristic pathological manifestations such as amyloidosis, immunotactoid glomerulopathy, type I cryoglobulinemic glomerulonephritis, Randall-type monoclonal immunoglobulin disease, proliferative glomerulonephritis with monoclonal IgG deposits, and light chain proximal tubulopathy [19]. The present case, along with a similar case reported previously [13], further emphasizes that the presence of C3 glomerulopathy, including DDD, should be recognized as an incipient manifestation of MGRS. Although the current case did not show the typical morphological features of classic DDD, a previous case report described similar ultrastructural findings in an older patient with monoclonal gammopathy [13]. Because of the limited number of published cases, it is not clear that DDD related to monoclonal immunoglobulins generally demonstrates the distinct pathological findings of classic DDD. Further case studies are required to delineate the pathological characteristics of this rare disorder.

In the case of this patient who initially presented with DDD and was subsequently diagnosed with MGRS, BD therapy resulted in clinical improvement and histological amelioration of disease activity. This case highlights the need to increase awareness of DDD as a potential manifestation of MGRS in middle-aged and elderly patients. As illustrated in this case, rapid and efficient chemotherapy can limit renal damage in monoclonal gammopathy-associated DDD.

## Abbreviations

BD: Bortezomib and dexamethasone
C3: Complement component 3
DDD: Dense deposit disease
MGRS: Monoclonal gammopathy of renal significance
MGUS: Monoclonal gammopathy of unknown significance
MPGN: Membranoproliferative glomerulonephritis

## References

[1] Walport MJ (2001). Complement. First of two parts. N Engl J Med.

[2] Zipfel PF, Skerka C (2009). Complement regulators and inhibitory proteins. Nat Rev Immunol.

[3] West CD, McAdams AJ, MacConville JM (1965). Hypocomplementaemic and normocomplementaemic persistent (chronic) glomerulonephritis; clinical pathologic characteristics. J Pediatr.

[4] Bomback AS, Appel GB (2012). Pathogenesis of the C3 glomerulopathies and reclassification of MPGN. Nat Rev Nephrol.

[5] Pickering MC, D'Agati VD, Nester CM (2013). C3 glomerulopathy: consensus report. Kidney Int.

[6] Barbour TD, Ruseva MM, Pickering MC (2016). Update on C3 glomerulopathy. Nephrol Dial Transplant.

[7] Fakhouri F, Fremeaux-Bacchi V, Noel LH (2010). C3 glomerulopathy: a new classification. Nat Rev Nephrol..

[8] Servais A, Noel LH, Roumenina LT (2012). Acquired and genetic complement abnormalities play a critical role in dense deposit disease and other C3 glomerulopathies. Kidney Int.

[9] Sethi S, Zand L, Leung N (2010). Membranoproliferative glomerulonephritis secondary to monoclonal gammopathy. Clin J Am Soc Nephrol.

[10] Leung N, Bridoux F, Hutchison CA (2012). Monoclonal gammopathy of renal significance: when MGUS is no longer undetermined or insignificant. Blood.

[11] Merlini G, Stone MJ (2006). Dangerous small B-cell clones. Blood.

[12] Bridoux F, Desport E, Fremeaux-Bacchi V (2011). Glomerulonephritis with isolated C3 deposits and monoclonal gammopathy: a fortuitous association?. Clin J Am Soc Nephrol.

[13] Sethi S, Sukov WR, Zhang Y (2010). Dense deposit disease associated with monoclonal gammopathy of undetermined significance. Am J Kidney Dis.

[14] Jokiranta TS, Solomon A, Pangburn MK, Zipfel PF, Meri S (1999). Nephritogenic lambda light chain dimer: a unique human miniautoantibody against complement factor H. J Immunol.

[15] Zand L, Kattah A, Fervenza FC (2013). C3 glomerulonephritis associated with monoclonal gammopathy: a case series. Am J Kidney Dis.

[16] Sethi S, Fervenza FC (2012). Membranoproliferative glomerulonephritis--a new look at an old entity. N Engl J Med.

[17] Chapter 8 (2012). Idiopathic membranoproliferative glomerulonephritis. Kidney Int Suppl.

[18] Lorenz EC, Sethi S, Leung N, Dispenzieri A, Fervenza FC, Cosio FG (2010). Recurrent membranoproliferative glomerulonephritis after kidney transplantation. Kidney Int.

[19] Bridoux F, Leung N, Hutchison CA (2015). Diagnosis of monoclonal gammopathy of renal significance. Kidney Int.

